# Enhancement of peripheral fatty acyl ethanolamide signaling prevents stress-induced social avoidance and anxiety-like behaviors in male rats

**DOI:** 10.1007/s00213-023-06473-w

**Published:** 2023-11-07

**Authors:** Luca Carnevali, Margherita Barbetti, Yannick Fotio, Francesca Ferlenghi, Federica Vacondio, Marco Mor, Daniele Piomelli, Andrea Sgoifo

**Affiliations:** 1 Stress Physiology Lab, Department of Chemistry, Life Sciences and Environmental Sustainability, University of Parma, Parma, Italy; 2 Department of Anatomy and Neurobiology, University of California, Irvine, CA 92697 USA; 3 Department of Food and Drug, University of Parma, Parma, Italy; 4 Department of Pharmaceutical Sciences, University of California, Irvine, CA 92697 USA; 5 Department of Biological Chemistry, University of California, Irvine, CA 92697 USA

**Keywords:** Stress, FAAH, URB937, Anxiety, Fatty acyl ethanolamide, Cytokines

## Abstract

**Rationale:**

Exposure to traumatic events can lead to alterations in social and anxiety-related behaviors. Emerging evidence suggests that peripheral host-defense processes are implicated in the expression of stress-induced behavioral responses and may be targeted to mitigate the negative sequalae of stress exposure.

**Objectives:**

In this study, we used the peripherally restricted FAAH inhibitor URB937 to investigate the effects of the fatty acyl ethanolamide (FAE) family of lipid mediators – which include the endocannabinoid anandamide and the endogenous PPAR-α agonists, oleoylethanolamide and palmitoylethanolamide – on behavioral and peripheral biochemical responses to two ethologically distinct rat models of stress.

**Methods:**

Male adult rats were exposed to acute social defeat, a model of psychological stress (Experiment 1), or to the predator odor 2,5-dihydro-2,4,5-trimethylthiazoline (TMT), a test of innate predator-evoked fear (Experiment 2), and subsequently treated with URB937 (1 or 3 mg/kg, intraperitoneal) or vehicle. Behavioral analyses were conducted 24 h (Experiment 1) or 7 days (Experiment 2) after exposure.

**Results:**

URB937 administration prevented the emergence of both social avoidance behavior after social defeat stress and anxiety-related behaviors after TMT exposure. Further, URB937 administration blocked social defeat-induced transient increase in plasma concentrations of pro-inflammatory cytokines and the elevation in plasma corticosterone levels observed 24 h after social defeat

**Conclusions:**

Enhancement of peripheral FAAH-regulated lipid signaling prevents the emergence of stress-induced social avoidance and anxiety-like behaviors in male rats through mechanisms that may involve an attenuation of peripheral cytokine release induced by stress exposure.

**Supplementary Information:**

The online version contains supplementary material available at 10.1007/s00213-023-06473-w.

## Introduction

Exposure to traumatic events initiates a cascade of neural responses which, in vulnerable individuals, can result in the development of post-traumatic stress disorder (PTSD), anxiety, and other disease conditions (Fekadu et al. 2019; Shepherd and Wild 2014). In addition to central processes, peripheral mechanisms have emerged as an important component of the physio-pathological reaction to stress. Such mechanisms could be targeted to mitigate the negative sequelae of stress exposure.

A possible pharmacological strategy is to enhance the protective effects of the fatty acyl ethanolamide (FAE) family of bioactive lipid mediators. The FAEs include endogenous agonists of cannabinoid receptors (e.g., anandamide) and peroxisome proliferator-activated receptor-α (e.g., palmitoylethanolamide (PEA) and oleoylethanolamide (OEA)). FAE-mediated signaling is terminated by the intracellular serine hydrolase, fatty acid amide hydrolase (FAAH) (McKinney and Cravatt 2005). Relatedly, preclinical studies have shown that globally active FAAH inhibitors such as URB597 (Kathuria et al. 2003) increase availability of endogenous FAEs and promote behavioral resilience to stress through mechanisms that have generally been ascribed to central processes, including enhanced monoaminergic neurotransmission and increased neurogenesis in the hippocampus (Carnevali et al. 2017; Danandeh et al. 2018; Gobbi et al. 2005). Yet, FAEs-mediated signaling also impacts peripheral processes as shown, for example, by the marked analgesic and anti-inflammatory properties of peripherally restricted FAAH inhibitors such as URB937 (Mabou Tagne et al. 2022; Piomelli and Sasso 2014).

Notably, pro-inflammatory cytokines are emerging as important peripheral mediators of the pathological consequences of psychosocial stress (Hodes et al. 2014; Niraula et al. 2019). For example, increases in circulating concentrations of pro-inflammatory cytokines and chemokines have been observed in patients with PTSD or anxiety as well as in rats and mice that exhibit anxiety-like behaviors following stress exposure (Cheng et al. 2015; Kalinichenko et al. 2014; Passos et al. 2015). Preclinical studies have also found that stress-induced peripheral expression of the pro-inflammatory cytokine interleukin-6 (IL-6) plays a significant role in the recruitment of monocytes to the brain and the development of behavioral abnormalities in mice exposed to repeated social defeat stress (Hodes et al. 2014; Niraula et al. 2019). Supporting a role for peripheral IL-6, acute treatment with a monoclonal IL-6 antibody, which does not enter the brain, prevented stress-induced social avoidance behavior (Hodes et al. 2014). Notably, the idea that peripheral cytokines might contribute to the development of stress-related behavioral alterations is underscored by the finding that “sickness behavior” – a term used to describe the abnormal behavior (i.e., social withdrawal) exhibited by many mammals when they are physically ill – may be triggered by pro-inflammatory cytokines produced at the site of injury and/or infection (Dantzer et al. 2008). Collectively, these studies suggest that peripheral cytokine release may contribute to the expression of abnormal behavioral responses to stress, which could be targeted by enhancing peripheral FAE-mediated signaling through FAAH inhibition.

In the present study, we exposed male rats to acute social defeat, a paradigm that captures key aspects of the human response to psychosocial stress (Huhman 2006; Koolhaas et al. 2013), or to the kairomone 2,5-dihydro-2,4,5-trimethylthiazoline (TMT), a test of innate predator-evoked fear (Takahashi et al. 2005). These ethologically distinct models are widely used to study the behavioral and biological consequences of trauma exposure in rodents (Verbitsky et al. 2020). In both cases, we inhibited peripheral FAAH activity after exposure to the stressor, using the selective, brain-impermeant FAAH inhibitor URB937 (Clapper et al. 2010; Moreno-Sanz et al. 2011). We tested the hypothesis that enhancement of peripheral FAE-mediated signaling promotes behavioral resilience in both models and explored potential associated changes in peripheral pro-inflammatory cytokine release.

## Materials and Methods

### General experimental design

**Fig. 1:**
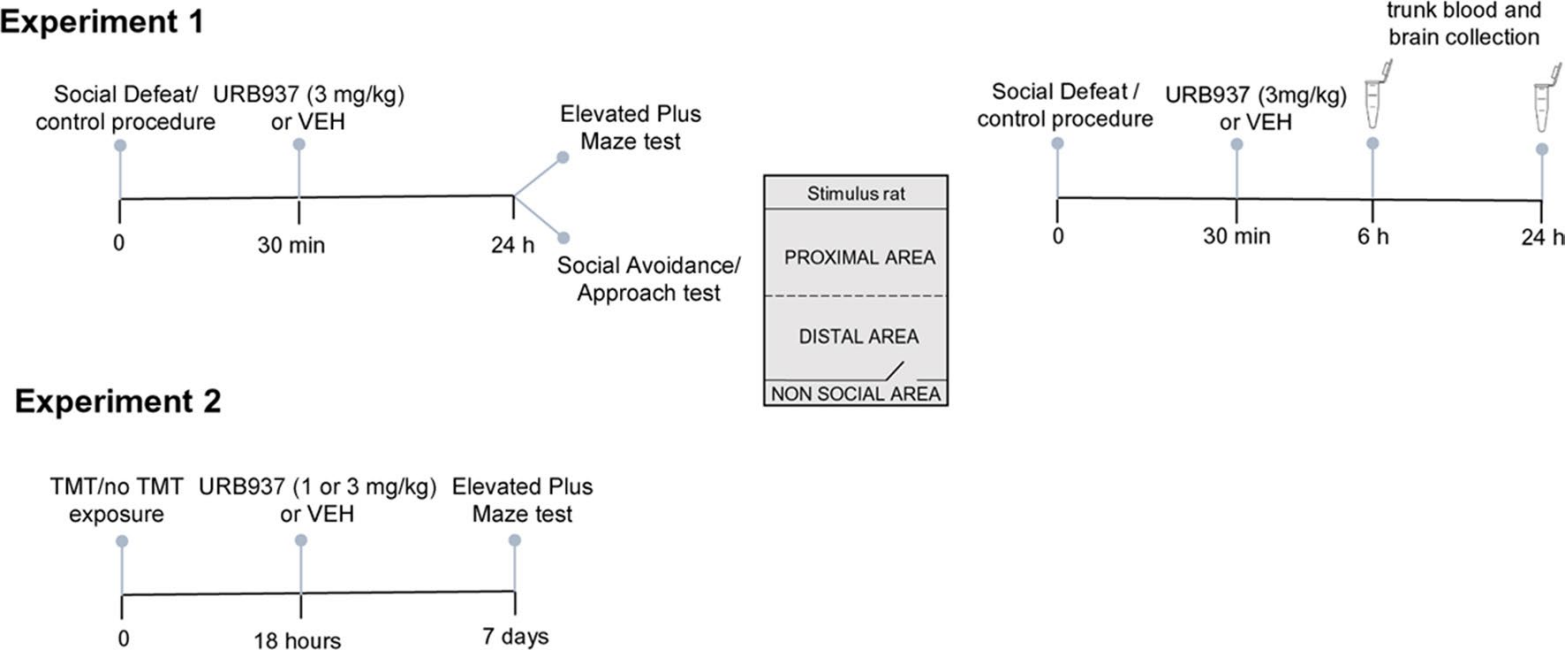
Timelines of experimental procedures. Abbreviations: TMT = 2,5-dihydro-2,4,5-trimethylthiazoline; VEH = vehicle

We administered URB937 at doses (1 and 3 mg/kg) known to cause profound FAAH inhibition in peripheral organs but no detectable effect on brain FAAH activity (Clapper et al. 2010; Vozella et al. 2019). In Experiment 1, which was conducted at the University of Parma (Italy), rats were randomly assigned to social defeat stress (SDS) or control (CTR) procedure, as outlined below. Thirty min later, the animals received a single injection of vehicle or URB937. This time point was chosen to allow the return of rats to their home cages after SDS/CTR procedure and based on previous studies showing that rats still show signs of potent SDS-induced sympathoadrenergic activation during this phase (e.g., Barbetti et al. 2022). On the following day, behavior was evaluated using either the social avoidance/approach (SAA) test or the elevated plus maze (EPM) test to determine whether peripheral FAAH inhibition might influence the development of short-term social avoidance and anxiety-like behaviors in rats exposed to SDS. A separate group of SDS and CTR rats was euthanized 6 and 24 h after the procedure, and blood and brains were collected to measure plasma and brain concentrations of proinflammatory cytokines and FAEs, and plasma concentrations of corticosterone. Experimental procedures were approved by the Italian legislation on animal experimentation (D.L. 04/04/2014 n. 26, authorization n. 449/2017-PR). In Experiment 2, which was conducted at the University of California Irvine (USA), rats were randomly exposed to TMT or saline, as outlined below. After 18 h, they received a single injection of vehicle or URB937 and were returned to their home cages. This time point was selected because it was previously shown that administration of the global FAAH inhibitor URB597 18 h after TMT exposure prevented the consolidation of anxiety-like behavior (EPM test) in rats (Danandeh et al. 2018). Seven days later rats were submitted to the EPM test to evaluate whether peripheral FAAH inhibition influenced the establishment of long-term anxiety-like behavior caused by TMT. All procedures met the National Institutes of Health guidelines for the care and use of laboratory animals and were approved by the Institutional Animal Care and Use Committee of the University of California, Irvine. For both experiments, analyses were performed under blinded conditions, and sample size was not predetermined. Behavioral tests were conducted between 9 and 10am. The study report follows ARRIVE guidelines (Kilkenny et al. 2010). The experimental protocols are illustrated in Fig. 1.

### Chemicals

URB937 (N-cyclohexyl-carbamic acid, 3'-(aminocarbonyl)-6-hydroxy[1,1'-biphenyl]-3-yl ester) was synthesized as described (Moreno-Sanz et al. 2014). TMT, a volatile constituent of fox feces that is innately aversive to some rodent species, was purchased from SRQ (Sarasota, FL). All other reagents and solvents were of the highest available grade and were purchased from Sigma-Aldrich (St. Louis, USA).

### Drug preparation

URB937 was freshly prepared by suspension into polyethylene glycol (PEG-400) and addition of an equal volume of Tween-80, as described (Vozella et al. 2019). The mixture was vortexed and sonicated to obtain a clear solution. Sterile saline was added, and the final solution (10% PEG-400, 10% Tween-80 and 80% saline) was sonicated for 5 min at 37 °C. URB937 and vehicle were administered by intraperitoneal (IP) injection in a volume of 1 ml/kg.

### Animals

Animals were kept in climate-controlled rooms (temperature: 22 °C and humidity: 50–60%). For Experiment 1, 3-month-old male Wistar rats (*n* = 120; bred at the University of Parma) were singly housed under a 12-h reverse light/dark cycle (lights on at 7 pm). Additional 6-month-old male wild-type Groningen rats, each housed with an oviduct-ligated female partner, were used as aggressive residents in the resident-intruder paradigm. For Experiment 2, 2-month-old male Sprague–Dawley rats (*n* = 33; Charles River, Wilmington, MA) were housed (4 per cage) under a 12-h light/dark cycle (lights on at 6:30am).

### Acute social defeat stress

The acute social defeat stress (SDS) model is based on a modified version of the classical “resident–intruder” paradigm (Koolhaas et al. 2013). Resident rats were screened for their aggressive behavior before the beginning of the procedure. After the removal of the resident’s female partner, each rat from the SDS group was placed in the cage of a resident rat, separated from the latter by a wire mesh partition. Fifteen min later, the partition was removed allowing physical interaction – consisting of repeated attacks by the aggressive resident and subordination of the intruder – for additional 15 min. On the same day but in a separate room, CTR rats were placed in a novel and empty cage for 30 min. After the procedure, all rats were returned to their home cages. Rats were closely inspected after SDS and none of them reported any improper injury or wound. The EPM/SAA tests were conducted 24 h after SDS.

### TMT exposure

We exposed rats to TMT or saline as described (Danandeh et al. 2018; Ivy et al. 2020). The animals were individually placed for 15 min in a plastic exposure box (30 × 22 × 22 cm) containing a square of gauze (5 × 5 cm) doused with sterile saline (50 μl). Exposure was conducted in a fume hood. On the day of the experiment, the rats were randomly selected and placed for 20 min in the box containing a gauze doused with 50 μl of either saline or TMT. Upon completion of the procedure, the animals were immediately returned to their cages. The EPM test was conducted one week after TMT exposure.

### Behavioral tests

The EPM test was carried out as described (Danandeh et al. 2018; Ivy et al. 2020). Each rat was placed on the central platform of the maze, facing an open arm, and behavior was recorded for 5 min using a Debut video capture software (NCH Software, Canberra, Australia). A blinded observer measured the amount of time spent in and the number of entries into the open and closed arms. The anxiety index was calculated as previously described (Fotio et al. 2021): 1-[(time spent in open arms/total time) + (open arm entries/total entries)]/2. Between tests, the apparatus was cleaned with an ethanol solution (20% in water).

The SAA test (Haller and Bakos 2002) was conducted as described (Carnevali et al. 2014). The experimental apparatus consisted of two chambers – a “non-social” (40 × 40 × 40 cm) and a “social” (20 × 40 × 40 cm) compartment – connected by a sliding door. The social chamber contained an enclosure (15 × 40 × 40 cm) delimited by a wire mesh partition in which a male stimulus-unfamiliar rat—of the same wild-type Groningen strain of the resident rats—was confined (Fig. 1). On the test day, the rats were individually placed in the non-social chamber for a 2-min habituation period. The sliding door was then opened, and the animals were allowed to move freely for 10 min. Behavior was recorded using a video camera positioned above the apparatus. At the end of the test, the apparatus was thoroughly cleaned. Behavior was scored by trained personnel blinded to the experimental condition. For analyses, the social chamber was divided into two zones of equal size – proximal to and distal from the stimulus rat – as described (Carnevali et al. 2014). The following parameters were measured: (i) time spent in the non-social compartment, in the proximal zone and in the distal zone of the social compartment (expressed as % of total time), and (ii) latency (expressed as seconds) to the first access to the social compartment.

### Biochemical measurements

Rats were euthanized 6 or 24 h after the SDS/CTR procedure, brains were removed, and trunk blood was collected into anticoagulated test tubes (Sarsted AG, Numbrecht, Germany). Plasma was immediately prepared by centrifugation (2,600 g; 4 °C; 10 min) and stored at -20 °C until analysis. Brains were stored at -80 °C until analysis. Brain concentrations of IL-6 and IL-1β were measured by solid-phase sandwich ELISA kits for rat tissue lysates following the manufacturer’s procedural guidelines (IL-6: catalog Reference: ERA32RB; IL-1: β catalog reference: ERIL1B; Thermo Fisher Scientific, Waltham, MA, USA). Plate absorbance was read at the wavelength of 450 nm employing a Tecan Spark 10 M Multimode Plate Reader (Tecan, Wien, Austria). Plasma concentrations of IL-6, IL-1β and TNF-α were measured using the ProcartaPlex™ Multiplex Immunoassay kit (Thermo Fisher Scientific, USA).

Corticosterone was quantified in rat plasma by high performance liquid chromatography (HPLC) coupled to tandem mass spectrometry (MS/MS) employing a previously published bioanalytical method (Carnevali et al. 2015). A detailed description of the method and instrumental configuration is reported under Supplementary Material.

A previously reported HPLC–MS/MS method was employed to quantify anandamide, OEA and PEA in brain tissue homogenates (10% w/v) and plasma (Carnevali et al. 2020, 2015). A detailed description of the HPLC–MS/MS method is reported in the Supplementary Material.

### Statistical analyses

Statistical analyses were conducted using IBM SPSS Statistics, version 27 (IBM Corp., Armonk, N.Y., USA). The normal distribution of variables was determined using the Kolmogorov–Smirnov test. Data were analyzed using 2 (“group”: stress or control condition) × 2 (“treatment”: URB937 at 1 or 3 mg/kg or VEH injection) factorial design analysis of variance, followed by pre-planned analyses using unpaired Student’s *t* test with a Bonferroni correction for multiple comparisons for each outcome variable separately. Statistical significance was set at p < 0.05.

## Results

### URB937 prevents avoidance behavior evoked by SDS

**Fig. 2:**
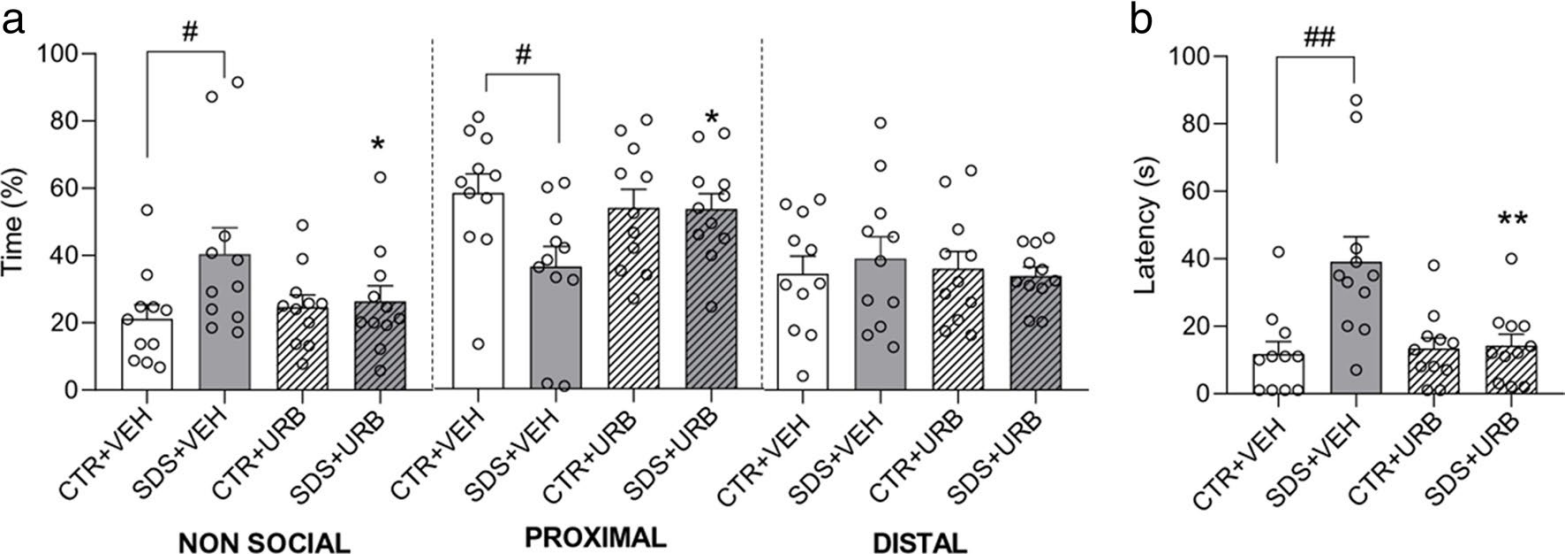
Behavior in the Social Approach/Avoidance test 24 h after social defeat stress/control procedure (*n* = 11 per group). (**a**) Time spent by experimental rats in the non-social and social (i.e., proximal to and distal to the stimulus rat, respectively) compartments of the social approach–avoidance apparatus. (**b**) Latency to the first access to the social compartment. Values are expressed as means (± SEM). Abbreviations: SDS = social defeat stress; CTR = control; VEH = vehicle; URB = URB937. ^#^ and ^##^ = *p* < 0.05 and *p* < 0.01, respectively; * and ** = *p* < 0.05 and *p* < 0.01, respectively, versus STR + VEH value

In Experiment 1, we subjected rats to acute SDS and evaluated their behavior 24 h later in the SAA and EPM tests (Fig. 1). SDS produced a marked social avoidance behavior in the SAA test, which was prevented by administration of URB937 (3 mg/kg) 30 min after SDS exposure (Fig. 2). ANOVA showed a significant stress x treatment interaction for the time spent in the non-social compartment (F_(1,40)_ = 3.9, p < 0.05) and in the proximal zone of the social compartment (F_(1,40)_ = 4.12, p < 0.05). Specifically, SDS + VEH rats spent more time in the non-social area and less time in the proximal area of the social compartment (p < 0.05) compared to CTR + VEH rats (Fig. 2a). Importantly, no such effects were observed in stressed rats treated with URB937 (SDS + URB). Subsequent analysis of the latency to first access the social compartment (Fig. 2b) yielded a significant stress x treatment interaction (F_(1,40)_ = 7.72, *p* < 0.01). SDS + VEH rats showed a longer latency to first enter the social area compared to CTR + VEH and SDS + URB animals (*p* < 0.01). On the other hand, SDS did not produce any significant effect on anxiety-like behaviors in the EPM test (Table 1). The results suggest that inhibition of peripheral FAAH activity after exposure to acute SDS stops the emergence of social avoidance behavior in male rats.

**Table 1:** Behavior in the Elevated Plus Maze test 24 h after social defeat stress/control procedure

	Time in open arms (s)	Time in closed arms (s)	Open arms entries (n)	Closed arms entries (n)	Anxiety index
CTR + VEH	41.7 ± 12.7	258.3 ± 12.7	2.0 ± 0.5	5.5 ± 0.9	0.79 ± 0.05
SDS + VEH	38.5 ± 10.1	261.5 ± 10.1	2.3 ± 0.6	8.2 ± 0.8	0.83 ± 0.03
CTR + URB	46.7 ± 6.8	253.3 ± 6.8	3.3 ± 0.2	6.3 ± 0.6	0.76 ± 0.03
SDS + URB	34.5 ± 5.5	265.5 ± 5.5	2.2 ± 0.3	5.5 ± 0.6	0.80 ± 0.03

The anxiety index was calculated as 1-[(time in the open arms/total time) + (open arm entries/total entries)]/2 and is reported as mean ± SEM (*n* = 7/group). Abbreviations: SDS = social defeat stress; CTR = control; VEH = vehicle; URB = URB937

### URB937 prevents the increase in plasma corticosterone evoked by SDS

**Fig. 3:**
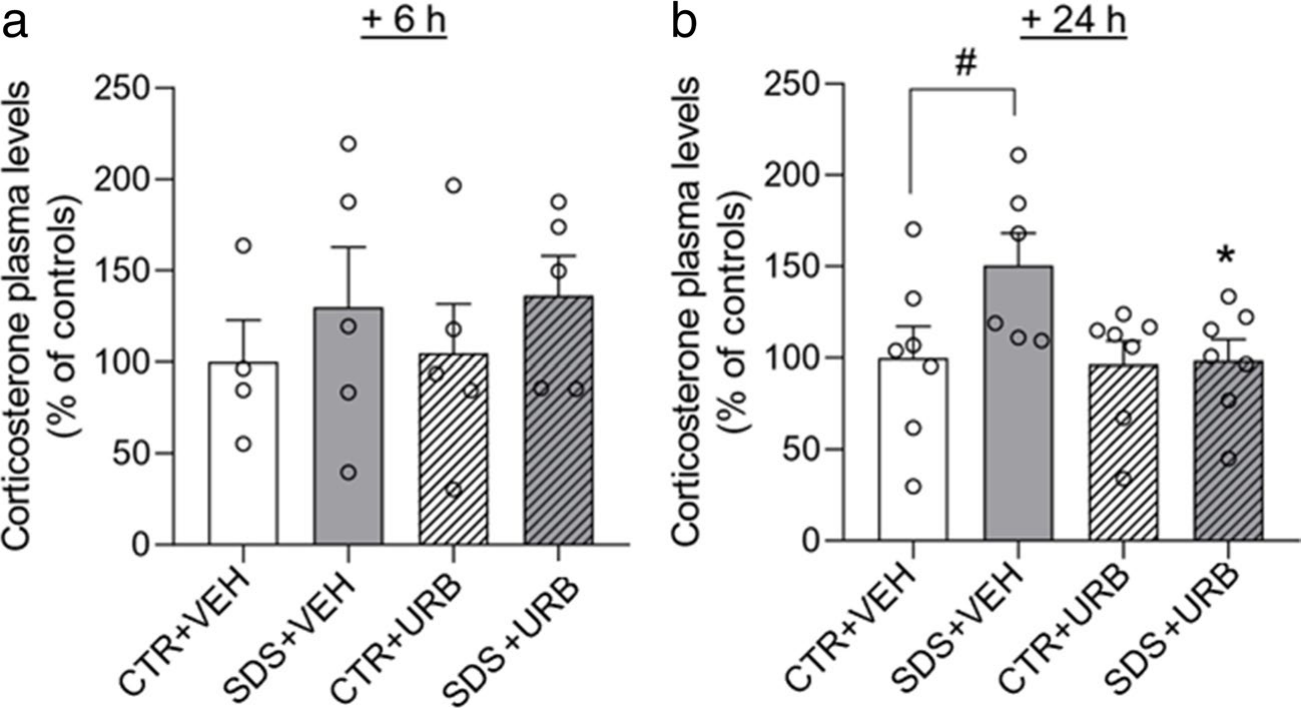
Plasma levels of corticosterone measured 6 h (*n* = 4/5 per group, panel **a**) and 24 (*n* = 6/7 per group, panel **b**) h after social defeat stress/control procedure. Values are expressed as means (± SEM). Abbreviations: SDS = social defeat stress; CTR = control; VEH = vehicle; URB = URB937. ^#^ = *p* < 0.05; * = *p* < 0.05 versus STR + VEH value

Next, we asked whether peripheral FAAH inhibition might also prevent the rise in circulating corticosterone evoked by acute SDS. Male rats were exposed to SDS/CTR procedure and were euthanized 6 h or 24 h later for analysis. ANOVA revealed a significant effect of pharmacological treatment (F_(1,23)_ = 4.18, *p* < 0.05) and a marginally significant effect of stress (F_(1,23)_ = 3.77, *p* = 0.06) at the 24 h time point. Plasma corticosterone levels were significantly higher in SDS + VEH rats compared with CTR + VEH rats (*p* < 0.05) (Fig. 3B). The effect of SDS was blocked by post-stress URB937 administration (Fig. 3b). Neither SDS nor URB937 affected plasma corticosterone at 6 h (Fig. 3a). We interpret these findings as indicating that inhibition of peripheral FAAH activity after acute SDS attenuates the delayed hormonal change caused by this procedure in male rats.

### URB937 prevents the increase in circulating cytokines evoked by SDS

**Fig. 4:**
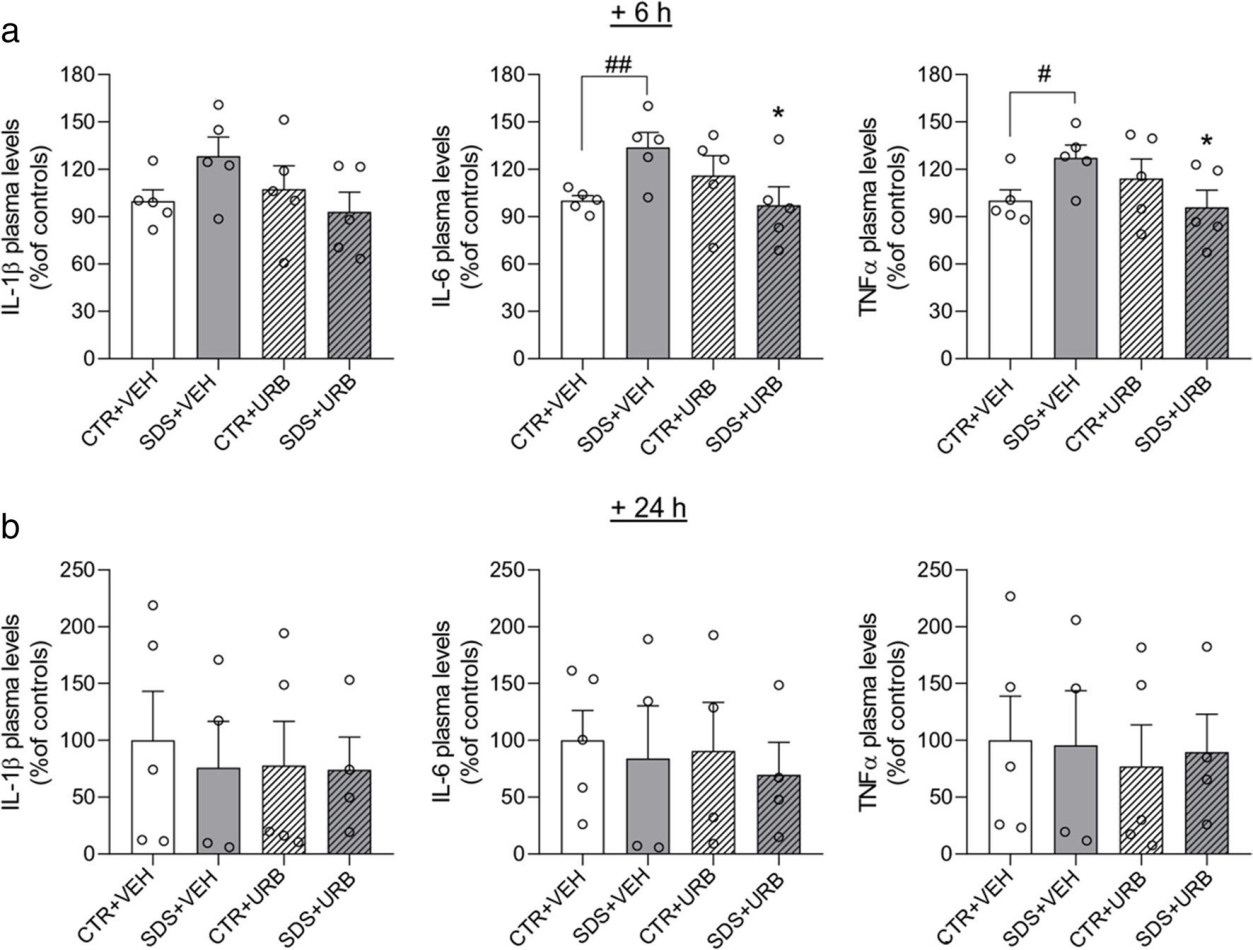
Plasma levels of IL-6, TNF-α and IL-1β measured 6 h (**a**) and 24 h (**b**) after social defeat stress /control procedure (*n* = 4/5 per group). Values are expressed as means (± SEM). Abbreviations: SDS = social defeat stress; CTR = control; VEH = vehicle; URB = URB937. ^#^ and ^##^ = *p* < 0.05 and *p* < 0.01, respectively; * = *p* < 0.05 versus STR + VEH value

The release of pro-inflammatory cytokines may contribute to the pathological consequences of psychosocial stress (Hodes et al. 2014; Niraula et al. 2019). We examined therefore whether post-stress administration of URB937 might affect the plasma concentrations of three representative cytokines – IL-β IL-6, and TNF-α – 6 and 24 h following acute SDS. Six hours after the challenge, plasma levels of IL-6 (p < 0.01) and TNF-α (p < 0.05) were significantly higher in SDS + VEH than CTR + VEH rats (Fig. 4a). Likewise, plasma levels of IL-1β trended higher in SDS + VEH than CTR + VEH rats, although this difference did not reach statistical significance (*p* = 0.07) (Fig. 4a). Cytokine levels effects were normalized by treatment with URB937 (Fig. 4A) and receded at the 24-h time point (Fig. 4b). SDS did not alter cytokine production in brain tissue (Table 2).

**Table 2:** Brain levels of pro-inflammatory cytokines after social defeat stress/control procedure

		+ 6 h	+ 24 h
IL-1β (% of controls)	CTR + VEH	100.0 ± 9.3	100.0 ± 8.1
	SDS + VEH	95.4 ± 6.3	92.4 ± 9.7
	CTR + URB	113.0 ± 12.0	88.4 ± 7.2
	SDS + URB	110.0 ± 22.7	86.1 ± 10.0
IL-6 (% of controls)	CTR + VEH	100.0 ± 5.9	100.0 ± 5.3
	SDS + VEH	97.6 ± 4.3	82.2 ± 14.7
	CTR + URB	108.3 ± 10.9	82.7 ± 7.4
	SDS + URB	111.7 ± 16.4	107.9 ± 3.4

Brain levels of IL-6 and IL-1β measured 6 h and 24 h after social defeat stress or control procedure (*n* = 5 per group). Values are expressed as means (± SEM). Abbreviations: SDS = social defeat stress; CTR = control; VEH = vehicle; URB = URB937

### URB937 inhibits peripheral, not central FAAH activity

**Fig. 5:**
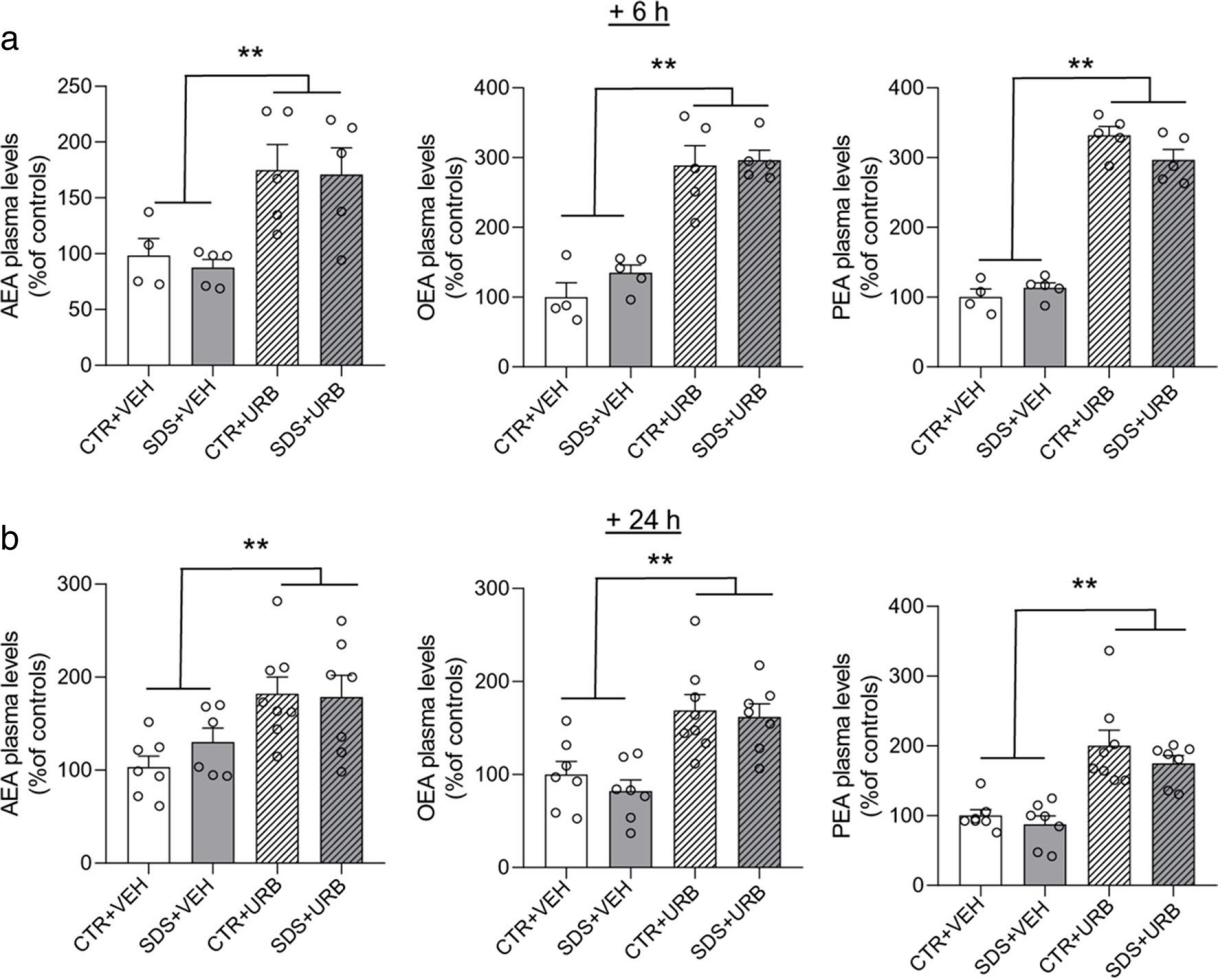
Plasma levels of fatty acyl ethanolamide 6 h (n = 4/5 per group, panel a) and 24 h (n = 6/7 per group, panel b) after social defeat stress/control procedure. Abbreviations: AEA = anandamide; OEA = oleoylethanolamide; PEA = palmitoylethanolamide. SDS = social defeat stress; CTR = control; VEH = vehicle; URB = URB937. Values are expressed as means (± SEM). ** = p < 0.01

Stress may affect the permeability of the blood–brain barrier (Menard et al. 2017) and might thus allow URB937, a substrate for the multi-drug transporters Abcg1 and Abcg2 (Moreno-Sanz et al. 2014, 2011), to access the CNS. To test this, we quantified anandamide, OEA and PEA in plasma and brain of SDS-exposed and CTR rats 6 h and 24 h after the stress challenge. The results indicate that post-stress administration of URB937 selectively increased concentrations of the three analytes in plasma (Fig. 5) but not in brain (Table 3). Two-way ANOVA showed a significant effect of treatment on circulating anandamide (F_(1,15)_ = 17.1, p < 0.01), OEA (F_(1,15)_ = 82.1, p < 0.01), and PEA (F_(1,15)_ = 307.9, p < 0.01) 6 h after SDS (Fig. 5a), whereas no such change was seen in the brain (Table 3). As expected for this covalently acting agent, the effect of URB937 were still detectable 24 h after administration (anandamide: F_(1,24)_ = 12.5, p < 0.01; OEA: F_(1,24)_ = 25.9, p < 0.01; PEA: F_(1,24)_ = 38.1, p < 0.01) (Fig. 5b). Of note, exposure to acute SDS did not significantly affect FAE levels in plasma (Fig. 5) or brain tissue (Table 3). Ex vivo enzyme activity measurements in brain and liver homogenates confirmed that URB937 selectively inhibited peripheral but not central FAAH activity (Table 4).

**Table 3:** Brain fatty acyl ethanolamide levels after social defeat stress/control procedure

		+ 6 h	+ 24 h
AEA (% of controls)	CTR + VEH	100.0 ± 8.8	100.0 ± 8.1
	SDS + VEH	88.1 ± 8.1	100.5 ± 7.3
	CTR + URB	112.4 ± 11.6	97.6 ± 10.7
	SDS + URB	105.3 ± 12.4	112.6 ± 8.2
OEA (% of controls)	CTR + VEH	100.0 ± 11.6	100.0 ± 4.5
	SDS + VEH	98.4 ± 9.1	116.7 ± 7.1
	CTR + URB	105.4 ± 8.5	98.5 ± 7.1
	SDS + URB	94.0 ± 6.2	123.3 ± 8.4
PEA (% of controls)	CTR + VEH	100.0 ± 7.0	100.0 ± 8.2
	SDS + VEH	94.9 ± 11.7	118.7 ± 11.9
	CTR + URB	98.9 ± 4.5	97.7 ± 9.2
	SDS + URB	105.3 ± 8.0	127.5 ± 11.7

Brain levels of anandamide, OEA and PEA measured 6 h and 24 h after social defeat stress or control procedure (*n* = 6–8 per group). Values are expressed as means (± SEM). Abbreviations: PEA = palmitoylethanolamide; AEA = anandamide; OEA = oleoylethanolamide; SDS = social defeat stress; CTR = control; VEH = vehicle; URB = URB937

**Table 4:** FAAH activity in brain and liver homogenates after social defeat stress/control procedure

		Brain		Liver
		+ 6 h	+ 24 h	+ 24 h
FAAH activity (% control)	CTR + VEH	100.0 ± 1.0	100.0 ± 8.8	100.0 ± 8.0
	SDS + VEH	99.7 ± 1.0	89.0 ± 6.5	82.9 ± 4.8
	CTR + URB	99.0 ± 1.8	71.9 ± 3.5	17.2* ± 4.3
	SDS + URB	98.8 ± 1.7	89.6 ± 9.1	22.5* ± 5.4

Values are reported as means ± SEM (n = 6/8 per group). Abbreviations: FAAH = fatty acid amide hydrolase; SDS = social defeat stress; CTR = control; VEH = vehicle; URB = URB937. * = p < 0.05 versus corresponding VEH value

### URB937 prevents the emergence of anxiety-like behaviors after TMT exposure

**Fig. 6:**
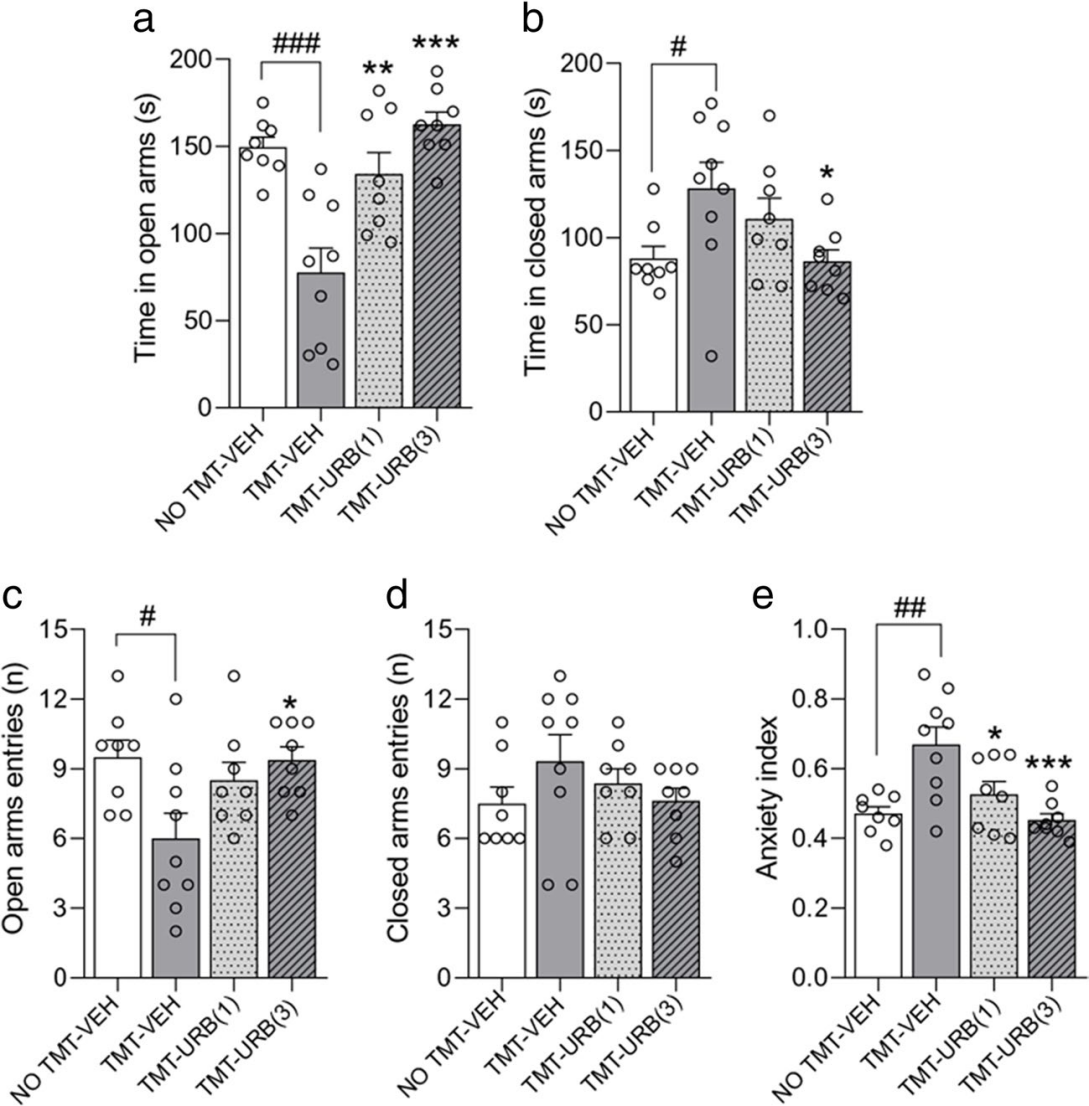
Behavior on the EPM test 7 days after TMT/saline exposure (*n* = 8/9 per group). Abbreviations: TMT = 2,5-dihydro-2,4,5-trimethylthiazoline; VEH = vehicle; URB = URB937 (1 or 3 mg/kg). Values are expressed as means (± SEM). ^#^, ^##^ and ^###^ = *p* < 0.05, *p* < 0.01 and *p* < 0.001, respectively; * and *** = *p* < 0.05 and *p* < 0.001 versus TMT-Veh

Finally, to assess the effects of peripheral FAAH inhibition in a model ethologically distinct from acute SDS, we challenged rats with the volatile fox kairomone TMT (Takahashi et al. 2005) and randomly assigned them to receive URB937 (1 or 3 mg/kg) or vehicle 18 h later. Anxiety-like behaviors were assessed 7 days after TMT exposure using the EPM test. ANOVA showed significant effects of TMT exposure (time in open arms: F_(1,29)_ = 23.1, p < 0.01; open arms entries: F_(1,29)_ = 9.1, p < 0.01; anxiety index: F_(1,29)_ = 16.3, p < 0.01) and URB937 treatment (time in open arms: F_(2,29)_ = 16.9, p < 0.01; open arms entries: F_(2,29)_ = 4.6, p < 0.05; anxiety index: F_(2,29)_ = 10.2, p < 0.01). Specifically, TMT-exposed rats treated with VEH spent significantly less time in the open arms (p < 0.05, Fig. 6a) and more time in the closed arms (p < 0.05, Fig. 6b) of the EPM, and entered open arms less frequently compared to control (i.e., NO-TMT) rats (p < 0.05, Fig. 0.6c). Consequently, the anxiety index was significantly greater in TMT-VEH rats compared to controls (p < 0.05, Fig. 6d). Importantly, URB937 administration after TMT exposure partially (1 mg/kg) or completely (3 mg/kg) abolished these behavioral effects (Fig. 6), suggesting that post-stress inhibition of peripheral FAAH activity prevents the development of anxiety-like behaviors in male rats exposed to TMT.

## Discussion

In the present study, we investigated the contribution of peripheral FAAH-regulated FAE signaling to the response to social defeat stress and TMT exposure, two ethologically distinct rodent models that are known to induce behavioral and biological phenotypes related to trauma exposure (Verbitsky et al. 2020). In both cases, we inhibited peripheral FAAH activity after exposure to the stressor by administering the brain-impermeant inhibitor URB937 (Clapper et al. 2010; Vozella et al. 2019), whose selectivity for FAAH outside the CNS was verified by LC/MS–MS. The results show that treating male rats with URB937 after stress (a) normalized both social behavior and circulating levels of corticosterone and pro-inflammatory cytokines following social defeat, and (b) attenuated persistent anxiety-like behavior caused by exposure to the predator odor TMT. We interpret these findings as indicating that peripheral FAAH-regulated FAE signaling exerts a previously unrecognized modulatory influence on behavioral responses to acute traumatic/stressful events, suggesting a pharmacological route outside the CNS to enhance behavioral resilience to stress.

### Behavioral and biochemical effects of social defeat stress and TMT exposure

The development of social avoidance 24 h after social defeat stress was not paralleled by signs of generalized anxiety-like behavior, as assessed by the EPM test. This finding is in line with previous work in mice showing that repeated social defeat disrupts social behavior but does not affect EPM performance (Hodes et al. 2014). This likely reflects the fact that the EPM test is more suitable for capturing general anxiety-like behavior than the SAA test (Walf and Frye 2007), which models anxiety-like behavior in an environmental/social context that reevokes the previous exposure to social defeat (Haller and Bakos 2002). Importantly, one of the most recognized symptoms of PTSD in humans is the avoidance of stimuli associated with traumatic event exposure (American Psychiatric Association 2013). Relatedly, the persistence of anxiety-like behavior seven days after TMT exposure replicates previous findings (e.g., Fotio et al. 2023) and has been related to fear extinction deficits, which characterize psychopathologies such as PTSD (Verbitsky et al. 2020). Therefore, signs of social avoidance behavior after social defeat stress and persistent anxiety-like behavior after exposure to predator scent in male rats may recapitulate the behavioral changes that predispose an individual specifically to the development of PTSD.

Rats exposed to a single episode of social defeat exhibited a transient increase in plasma concentrations of pro-inflammatory cytokines (IL-6, TNF-α, IL-1β), which was evident 6 h after social defeat. The 6-h interval was chosen based on previous work in mice showing that certain cytokines (including those measured here) reach peak levels between 6 and 12 h after a stressful event (Cheng et al. 2015). These findings are consistent with other studies documenting an increase in peripheral pro-inflammatory cytokines after acute and chronic stress exposure in rodents, and after acute psychological stress in healthy humans (Ando et al. 1998; Marsland et al. 2017). On the other hand, elevated plasma corticosterone levels were found in stressed rats 24 h but not 6 h after social defeat, which may be indicative of prolonged activation of the hypothalamic–pituitary–adrenal (HPA) axis after social defeat stress. The reason why corticosterone levels did not significantly differ at 6 h between SDS-exposed and CTR rats are unknown, but they might be ascribed to the stress experienced also by CTR animals after exposure to a novel cage and i.p. injection. Of note, other studies have reported a temporal dissociation between general HPA axis stress responses and stress-induced increases in proinflammatory cytokines as in the present investigation (e.g., Hodes et al. 2014).

Importantly, elevated peripheral markers of inflammation have been described in patients with PTSD and anxiety disorders (Cheng et al. 2015; Kalinichenko et al. 2014; Passos et al. 2015) and rodent studies strongly suggest that peripheral inflammatory cells play a causative role in the establishment of anxiety- and depressive-like symptoms (Hodes et al. 2014; Niraula et al. 2019; Wohleb et al. 2014a). The CNS has historically been viewed as an immune-privileged organ, in which adaptive immunity and inflammation are tightly controlled. However, peripheral mediators can influence CNS function, and potentially behavior, in several ways: for example, cytokines can cross the blood–brain barrier (Banks et al. 1991, 1994), activate primary afferent nerves (e.g., vagal nerve; Bluthe et al. 1994) or indirectly promote both microglia activation and myeloid cells recruitment to the brain (Engler et al. 2008; Wohleb et al. 2014a). Even though in our study brain cytokines levels did not differ between control and socially stressed rats, evidence from various rodent models suggests that monocyte trafficking to the brain promotes the development of anxiety-like behaviors following stress exposure (Wohleb et al. 2014a). It is tempting to speculate that elevated cytokine levels might have played a similar role also in our experiments. While we do not provide direct evidence to support or refute this hypothesis, the cause-effect relationship between monocyte trafficking to the brain and anxiety-like behavior has been documented in several preclinical studies. For example, inhibition of monocyte egress from bone marrow prevented the development of anxiety-like behavior in mice exposed to repeated social defeat (Engler et al. 2008; Wohleb et al. 2011). Similarly, monocyte trafficking to the brain was not observed in stressed mice lacking the IL-1 receptor type-1 and showing a behavioral resilient phenotype (Wohleb et al. 2014b).

### Effects of peripheral FAAH inhibition with URB937 after exposure to a traumatic/stressful event

Previous studies have shown that globally active FAAH inhibitors such as URB597 enhance behavioral resilience in rodent models of acute and chronic stress and exhibit profound anxiolytic- and antidepressant-like properties, which have been attributed – based on pharmacological, genetic, and biochemical data – to increased anandamide availability and heightened activation of CB_1_ cannabinoid receptors in stress-controlling circuits of the CNS (Bortolato et al. 2007; Carnevali et al. 2017; Gobbi et al. 2005; Kathuria et al. 2003). The current study provides the first evidence that administration of the brain-impermeant FAAH inhibitor URB937 immediately after exposure to a traumatic/stressful event (i) normalizes social behavior after SDS and anxiety-like behavior after TMT exposure, and (ii) blunts the SDS-dependent rise in circulating levels of corticosterone and pro-inflammatory cytokines. Further, confirming prior work (Clapper et al. 2010; Moreno-Sanz et al. 2011), URB937 increased the levels of three functionally significant FAEs – the endocannabinoid anandamide and the endogenous PPAR-α agonists OEA and PEA – in plasma and not in the brain. These results have three important implications: first, they suggest that peripheral mechanisms contribute to the behavioral response to stress; second, they suggest that the widely documented effects of globally active FAAH inhibitors on behavioral adaptations to stress may, at least partly, be ascribed to their peripheral action; third, they warrant further investigation on the utility of enhancing peripheral FAE levels as a strategy to counteract the negative behavioral consequences of stress.

An important question raised by the present data pertains to the specific mechanism through which peripheral FAE signaling modulates the response to stressful events. Two non-exclusive scenarios are especially plausible. Enhanced anandamide-mediated activation of CB_1_ receptors on peripheral noradrenergic nerve endings might dampen sympathetic outflow (Martinez-Torres et al. 2023; Pakdeechote et al. 2007), which is expected to impact the release of corticosterone and pro-inflammatory cytokines (Janig 2014). Alternatively, or additionally, enhanced PEA/OEA-mediated stimulation of PPAR-α - which are highly expressed in monocytes, macrophages, and other innate immune cells (Grabacka et al. 2021) – might suppress the peripheral reaction to stress. Contextually, it is important to point out that (i) circulating PEA levels are decreased in persons with PTSD (Hauer et al. 2013) as well as in healthy subjects experiencing a short-term depressed mood (Darmani et al. 2005); (ii) PEA adjunctive therapy to citalopram improves symptoms in patients with depression (Ghazizadeh-Hashemi et al. 2018); and (iii) intense physical activity improves depression and PTSD symptoms while concomitantly elevating plasma PEA levels (Heyman et al. 2012).

### Limitations

The two sets of experiments reported here were each conducted in a different laboratory. The reader will note that there are substantial experimental differences between the two, including design of the study protocols, strain of rats, and testing conditions. These differences were intentional, however, and in our view, they strengthen the finding that enhancement of peripheral FAE-dependent signaling promotes behavioral resilience to acute stress independently from contextual factors. Nevertheless, we acknowledge that others may see them as a limitation. Also, as pointed out above, we did not elucidate the molecular mechanism(s) through which peripheral FAE-dependent signaling may modulate the response to stress and we did not establish a causative link between cytokine levels and behavioral readouts. Relatedly, we did not assess biochemical parameters in the TMT model. Finally, we focused our investigation on male rats and thus cannot generalize our findings to female rats or other animal species, including humans.

## Conclusions

The present report provides the first evidence that peripheral FAAH-regulated lipid signaling protects male rats from the behavioral and biochemical consequences of acute stress, thus suggesting that pharmacological inhibition of FAAH activity outside the CNS might offer a new approach to the prevention of PTSD and other trauma-related diseases. These results are novel and relevant to our understanding of the potential contribution of peripheral processes in the normal and abnormal reactions to environmental challenges and to the discovery of innovative pharmacological strategies to foster behavioral resilience to stress.

## Supplementary Information

Below is the link to the electronic supplementary material.

Supplementary file1 (DOCX 31 KB)

## Data Availability

Data will be made available upon reasonable request.
