# Supplementary material for: Enhancement of peripheral fatty acyl ethanolamide signaling prevents stress-induced social avoidance and anxiety-like behaviors in male rats
Source: Psychopharmacology (Berl). 2023 Nov 7;242(5):997–1009. doi: 10.1007/s00213-023-06473-w (PMC12043783; doi:10.1007/s00213-023-06473-w)
Supplement: Supplementary file 1 — Supplementary file1 (DOCX 31 KB) [file 213_2023_6473_MOESM1_ESM.docx]

**Supplementary Material**

**MATERIALS AND METHODS**

**FAAH activity in rat brain and liver homogenates**

Ex vivo determination of FAAH activity in brain and liver tissue was carried out as previously reported (PMID: 19637155). [^3^H]-AEA (specific activity: 60 Ci/mmol), employed as substrate for ex vivo FAAH assay, was supplied by American Radiolabeled Chemicals (ARC Inc., St. Louis, MI, USA).

Briefly, rat brain and liver tissue was weighted and homogenized in 10 volumes of 50 mM ice-cold Tris buffer, pH 7.4, containing 0.32 M sucrose. Resulting homogenates were centrifuged (1,000 X g; 10 min; 4°C) and total protein content was quantified in the supernatant by the bicinchoninic acid (BCA) protein kit (Pierce Biotechnology, Rockford, IL, USA).

FAAH activity was measured in Tris buffer (0.5 ml, 50 mM, pH 7.5) at 37°C in the presence of 0.05% w/v fatty acid-free bovine serum albumin (BSA), 50 μg of protein from brain or 10 μg of protein from liver homogenates, 10 μM AEA and [^3^H]-AEA (10000 disintegrations per minute). After 30 min, enzymatic reaction was quenched by the addition of 1 ml chloroform:methanol (1:1 v/v).

After centrifugation (2,000 X g; 10 min; 4°C), [^3^H]-ethanolamine was measured in the aqueous phase by liquid scintillation counting.

**HPLC-MS/MS quantification of corticosterone in rat plasma**

Corticosterone (CORT) was quantified in rat plasma employing a previously developed HPLC-MS/MS bioanalytical method. Calibration curves were built in the concentration range 1500-10 nM by spiking charchoal treated rat plasma, pooled from control animals, with serially diluted stock solutions of CORT in DMSO (final DMSO concentration = 1%). Both calibration and unknown plasma samples were processed by addition of a double volume of ice-cold acetonitrile containing the structural analogue dexamethasone (DEXA) as internal standard (IS) at the concentration of 75 nM, centrifuged (16,000 X g, 10 min, 4 °C) and the supernatant directly analyzed by HPLC-MS/MS. The LOQ was equal to 10 nM for CORT. Calibration curves showed good linearity with coefficients of correlation (r^2^) > 0.99. Compound-dependent parameters were optimized by flow injection analysis of 5 μM standard solutions of CORT and IS DEXA in methanol. HPLC-MS/MS analysis was carried out in positive ion (ESI+) and in multiple-reaction monitoring (MRM) mode. The following parent ion → product ion transitions were monitored: CORT: m/z 347.1 [M+H]^+^ → m/z 105.1 + m/z 121.1 + m/z 329.2 (TL: 106 V; CE: 32; 24; 13 eV); DEXA: m/z 435.2 [M+H]^+^→ m/z 291.1 + m/z 309.2 + m/z 319.1 (TL: 95 V; CE: 18; 10; 11 eV). A Phenomenex Synergi Fusion Reverse Phase column (100x2.0 mm, 4 μm particle size; Phenomenex Srl, Italy) was employed for gradient separation at a flow rate of 0.35 ml/min. Eluent A: acetonitrile + 0.1% v/v formic acid; eluent B: water + 0.1% v/v formic acid. Gradient conditions: t(0 min): 5%A:95%B; t(1 min): 5%A:95%B; t(6 min): 100%A:0%B; t(11 min): 100%A:0%B; t(12 min): 5%A:95%B, with a 3-min equilibration time; total run time: 15 min. Injected volume: 10μl.

**HPLC-MS/MS quantification of fatty acid ethanolamides in rat brain and plasma**

The fatty acid ethanolamides AEA, OEA and PEA were extracted from 10% w/v brain tissue homogenates by protein precipitation via acetonitrile addition, as previously reported (PMID: 26391492; 31948828). Briefly, brain tissue was weighted and homogenized in Tris buffer (50 mM, pH 7.5, 0.32 M sucrose) to get a 10% w/v homogenate. For quantitative analysis, calibration curves were built in the 500-0.5 nM concentration range by spiking charcoal-treated rat plasma (pooled, from control animals) with freshly prepared stock solutions of AEA, OEA and PEA, serially diluted in DMSO (final DMSO concentration = 1%). Both calibration standards and unknown brain and plasma samples were processed by adding two volumes of ice-cold acetonitrile, containing 100 nM AEA-d_4_, OEA-d_4_ and PEA-d_4_ as internal standards, and, after a centrifugation step (16,000 X g; 10 min; 4°C), levels of fatty acid ethanolamides were quantified by HPLC–MS/MS. Calibration curves showed good linearity with coefficients of correlation (r^2^) > 0.99. The LOQ was equal to 0.5 nM for AEA, 2.5 nM for OEA and PEA. Compound-dependent parameters were optimized by flow injection analysis of 5 μM standard solutions in methanol. Acquisition occurred in positive ion (ESI+) and in multiple-reaction monitoring (MRM) mode. For quantitative analysis, the following parent ion → product ion transitions were selected: AEA: m/z 348.2 [M+H]^+^→ m/z 62.1 + m/z 90.9 Tube Lens (TL): 54 V; Collision Energy (CE): 14; 42 eV; AEA-d_4_: m/z 352.2 [M+H]^+^→ m/z 66.1 + m/z 202.8 + m/z 269.9 (TL: 76 V; CE: 17; 12; 19 eV); OEA: m/z 326.3 [M+H]^+^ → m/z 309.3 + m/z 93.3 + m/z 62.3 (TL: 115 V; CE: 10, 19 and 28 eV); OEA-d_4_: m/z 330.1 [M+H]^+^ → m/z 313.4 + m/z 66.2 (TL: 67 V; CE:14 and 15 eV); PEA: m/z 300.3 [M+H]^+^ → m/z 62.3 (TL: 54 V; CE:14 and 42 eV); PEA-d_4_: m/z 304.2 [M+H]^+^ → m/z 287.1 + m/z 66.3 (TL: 72 V; CE: 12 and 15 eV). A Waters XSelect HSS T3 column (100x2.1 mm, 3.5 μm particle size; Waters Corp, USA) was employed for separation employing a linear gradient at a flow rate of 0.22 ml/min. Eluent A: acetonitrile+0.1% v/v formic acid; eluent B: water+0.1% v/v formic acid; t(0 min): 5% A:95% B; t(1 min): 5% A:95% B; t(6 min): 100%A:0% B; t(11 min): 100% A:0% B; t(12 min): 5% A:95% B, followed by a 3 min equilibration time; total run time: 15 min). Injected volume: 10μl.

**HPLC-MS/MS system configuration**

A Thermo Accela UHPLC gradient system coupled to a Thermo TSQ Quantum Max triple quadrupole mass spectrometer (Thermo Fisher, USA) equipped with a heated electrospray ionization (H-ESI) ion source was employed for the analysis of corticosterone in rat plasma samples and of fatty acid ethanolamides in brain and plasma samples. H-ESI source tune parameters were set as follows: probe middle (D) position; capillary temperature: 270 °C; spray voltage: 4.0 kV. Nitrogen was the nebulizing gas at the following pressures: sheath gas: 35 psi; auxiliary gas: 15 arbitrary units (a.u.). Argon was used as the collision gas at a pressure of approximately 1.5 mtorr. The software Xcalibur version 2.2 (Thermo, Madison, WI, USA) was employed for HPLC-MS/MS data acquisition and processing.
